# Takotsubo cardiomyopathy after the first electroconvulsive therapy regardless of adjuvant beta-blocker use: a case report and literature review

**DOI:** 10.3325/cmj.2018.59.307

**Published:** 2018-12

**Authors:** Sara Medved, Zvonimir Ostojić, Hrvoje Jurin, Vesna Medved

**Affiliations:** 1 Department of Psychiatry, University Hospital Centre Zagreb, Zagreb, Croatia; 2 University Clinic of Cardiovascular Diseases, University Hospital Centre Zagreb, Zagreb, Croatia

## Abstract

Takotsubo cardiomyopathy (TC) is a rare complication of electroconvulsive therapy (ECT), an effective and safe treatment for severe cases of depression and psychosis. There are reports on 16 patients who developed TC after ECT, and these were predominantly female patients treated with antidepressants for depressive disorder. We describe a case of a 40-year-old male patient, with a history of schizophrenia and heavy caffeine and nicotine use, treated for acute psychotic episode with haloperidol and clozapine. Propranolol was administered because of clozapine-induced tachycardia. After 8 weeks without therapeutic response, the patient was referred for standard ECT procedure, which included premedication and bifrontotemporal stimulation. Two hours later, the patient experienced gastric pain and had increased troponin and natriuretic peptide levels and ST-elevation. After inotrope and anticoagulant treatment and replacement of antipsychotics, the patient remained stable. Contrary to common opinion, previous adrenergic blockade in this patient did not prevent TC occurrence. TC pathophysiology remains unclear although it has been related to the burst of norepinephrine neurons. Psychosis has also been associated with catecholamine dysfunction, and excessive psychological stress with long-term norepinephrine dysfunction. Animal models have shown that ECT, clozapine, and nicotine and caffeine use could considerably increase catecholamine levels. Clinical understanding of rare cardiac ECT complications could improve early recognition of patients at risk for TC and ensure safe ECT protocols.

Takotsubo cardiomyopathy is a rare complication of electroconvulsive therapy (ECT), an effective and safe treatment for severe depression and psychosis (1). It has been suggested that catecholamines play a role in takotsubo cardiomyopathy development (2).

## Case report

A 40-year-old white man, severely addicted to nicotine and caffeine, without alcohol misuse history, was admitted to our psychiatry department in December 2017 due to positive psychotic symptoms and was prescribed haloperidol (30 mg/d), promazine (300 mg/d), and diazepam (30 mg/d). He and his family members denied the history of medical conditions, and no medical data on his earlier treatment were found in our hospital’s archives. Three weeks later, because of treatment resistance, haloperidol and promazine were discontinued, and clozapine was augmented to a final dose of 350 mg a day. On the sixth day of clozapine therapy, the patient developed tachycardia and was given propranolol (40 mg/d) for cardioprotection. As psychosis did not improve by the end of the week 8, ECT was indicated.

Informed consent and ethical approval for ECT application were obtained from the University Hospital Centre Zagreb. The patient also signed informed consent for medical data publication. Somatic and psychiatric pre-evaluation revealed no contraindications for ECT. The blood pressure was 110/70 mm Hg, heart rate 92/min, axillary temperature 36.0°C, and electrocardiogram (ECG) showed a sinus rhythm with intermediate axis without any abnormalities. ECT was first applied at week 10. Diazepam was discontinued. Atropine, propofol, and succinylcholine were administered as standard premedication. The electrical dose was titrated to the patient’s seizure threshold at 0.5-millisecond pulse width, 20-Hz frequency, 5.6-second stimulus duration, and 900-mA current using Thymatron® System IV (Somatics LLC, Chatham, IL, USA), and bifrontotemporal stimulation was applied. Two hours after ECT, the patient complained of gastric pain. He was pale and tachypnoic, without a palpable radial pulse. Initial laboratory tests showed the troponin T level of 1956 ng/L (reference range <14 ng/L) and N-terminal pro-b-type natriuretic peptide (NT-pro BNP) of 12 409ng/L (reference range <300 ng/L). ECG showed marked ST-elevation, with Q waves and deep symmetrical T wave inversion in anterior leads, along with QTc interval prolongation (525 ms), indicating possible acute anterior wall myocardial infarction. However, the patient denied chest pain and complained of shortness of breath. The echocardiographic exam revealed severely reduced left ventricle ejection fraction (15%), with only lateral wall contracting and a consequent apical thrombus. The patient was immediately transferred to the Coronary Care Unit, where he was treated with continuous intravenous infusion of dobutamine (4 µg/min/kg) and furosemide (250 mg/d). Beside these, loading doses of aspirin (300 mg) and clopidogrel (600 mg), and low molecular weight heparin in full dose, were administered as standard therapy for suspicious acute myocardial infarction before planned coronarography. Clozapine was discontinued, and haloperidol (30 mg/d) and diazepam (30 mg/d) were induced intramuscularly. The patient refused the recommended coronary angiography and was distrustful to all other proposed diagnostic procedures. After 24 hours, he was hemodynamically stable, and inotropic support was gradually stopped. Heart failure therapy, consisting of eplerenone (25 mg/d) and ivabradine (2 × 5 mg/d), was initiated. Serial echocardiographic examinations over the next 4 days revealed that the left ventricular systolic function completely normalized, and troponin T and NT-proBNP level considerably decreased (228 ng/L and 1190 ng/L, respectively). ECG showing sinus rhythm, rate 82/min with a vertical axis, did not indicate myocardial ischemia. Based on troponin T and NT-proBNP levels, echocardiographic findings, ECG, and clinical restitution, the patient was diagnosed with takotsubo cardiomyopathy. He was then transferred back to the psychiatric department. At that time, clopidogrel was discontinued as the diagnosis of myocardial infarction seemed unlikely. During the next two weeks, echocardiographic exams were unremarkable, and troponin T and NT-proBNP levels were within the reference range (6 ng/L and 69 ng/L, respectively). By the end of week 12, the patient was referred to a chronic psychiatric institution.

## Discussion

Our hypothesis was that in our patient clozapine and ECT induced catecholamine activity discordant with beta-blocker effect. Of the 16 reported ECT-induced takotsubo cardiomyopathy cases so far, only one was a young male patient treated with clozapine, but without concomitant beta-blocker therapy (Table 1) (2-16).

**Table 1:** Case studies about Takotsubo cardiomyopathy after the use of electroconvulsive therapy (ECT) ordered by the year of publishing.

	Sex	Ethnicity	Age	Diagnose	Main therapy	Concomitant therapy	Number of ECT applications	Comorbidities	Alcohol/tobacco/illicit drugs use
Zhu 1992 (6)	F	Unknown	77	Major depression	Antidepressant therapy	Unknown	Not specified, 1st?	No history of cardiac disease	Unknown
Eitzman 1994 (7)	F	Unknown	76	Severe depression	Unknown	Unknown	Unknown	No history of medical treatment	Unknown
Ring 1996 (8)	F	Unknown	41	Major depressive dissociative disorder	Paroxetine 80 mg daily, naltrexone 200 mg daily	Propranolol long-acting 800 mg, propranolol 30 mg four times daily	1st course; 1st session	Obesity	Unknown
O’Reardon 2008 (16)	F	Unknown	45	Major depressive disorder	Unknown	Warfarin	1st course: 3rd session	Deep vein thrombosis with multiple pulmonary emboli, obstructive sleep apnea, hypertension, Loeys-Dietz syndrome	None
Chandra 2009 (12)	F	Unknown	70	Depression, bipolar disorder	Unknown	Unknown	Unknown	Mitral valve prolapse repair	Unknown
Go 2009 (9)	F	Unknown	50	Major depression with suicidal ideation	Quetiapine, venlafaxine, mirtazapine, pregabalin	Ropinirole, metformin, insulin, enalapril, hydroxyzine, fexofenadine, aspirin, furosemide	Unknown course; 3rd session	Type 2 diabetes mellitus, hypertension, obesity, asthma, sleep apnea, degenerative joint disease	Unknown
Go 2009 (9)	F	Unknown	49	Bipolar disorder; a depressed state with psychotic features	Valproate, fluoxetine, haloperidol, quetiapine	Albuterol, fluticasone/salmeterol	Unknown	Asthma, obesity, dyslipidemia, stress incontinence, impaired glucose tolerance	Unknown
Kent 2009 (13)	F	White	71	The major depressive episode with psychotic features	Nortriptyline 25 mg daily, quetiapine 200 mg daily, lorazepam 0.5 mg twice daily	Metoprolol extended-release 100 mg twice daily, amlodipine 5 mg twice daily, Nonsteroidal anti-inflammatory medications	3rd course; 3rd session	Hypertension, costochondritis	Unknown
Satterthwaite 2009 (10)	F	White	72	Major depression with catatonia	Unknown	Unknown	1st course; 1st session	Unknown	Unknown
Beach 2010 (1)	F	White	52	Treatment-resistant depression	Fluoxetine 80 mg daily, olanzapine 5 mg daily	Topiramate 200 mg daily, gabapentin 800 mg daily	Not specified, 1st?	Migraine headaches, cholecystectomy	Occasional alcohol use
Serby 2010 (5)	F	White	90	The recurrent major depressive disorder	Duloxetine 60 mg daily	Ranitidine 150 mg daily, calcium carbonate 600 mg twice daily, multivitamin once daily	>100 courses of ECT; mECT* for more than five years; 3rd session	Hypertension (normotensive without medication)	Unknown
Celano 2011 (14)	F	Unknown	76	The recurrent major depressive disorder	Mirtazapine, olanzapine	Unknown	1st course; 11 sessions (1st mECT* session)	Multiple myelomas	Unknown
Binhas 2013 (11)	F	Unknown	85	Depression, anorexia	Unknown	Unknown	Unknown course; 3rd session	Unknown	Unknown
Grubisha 2014 (3)	M	Unknown	31	Schizoaffective disorder, pervasive developmental disease	Clozapine 350 mg daily	Zonisamide	2nd course; 50 treatments	Hypertension, seizure disorder	Unknown
Narayanan 2014 (15)	F	White	74	The recurrent major depressive disorder	Venlafaxine, mirtazapine, lithium, aripiprazole	Bisoprolol 2.5 mg/daily, lisinopril 20 mg/daily	2nd course; 1st session	Chronic obstructive pulmonary disease, hypertension	None
De Wolf 2015 (4)	F	Unknown	67	The recurrent major depressive disorder	Fluvoxamine 300 mg daily, mirtazapine 15 mg daily	Unknown	Unknown (2nd?); 24 sessions (mECT*)	Oropharyngeal cancer	Unknown

*mECT – maintenance electroconvulsive therapy.

Although ECT-related cardiac complications have been occasionally reported, takotsubo cardiomyopathy remains the least understood among them. Takotsubo cardiomyopathy is a transient cardiac syndrome that most often involves left ventricular apical akinesis and mimics acute coronary syndrome, showing ST-segment elevation and cardiac enzyme levels similar to myocardial infarction (17). However, in takotsubo cardiomyopathy cardiac angiography can confirm no significant coronary artery stenosis. Typical echocardiography reveals left ventricular apical ballooning (17). Several takotsubo cardiomyopathy causes have been proposed, including coronary artery ischemia and microvascular spasm (18). The spasm is induced by a sudden catecholamine increase since patients with takotsubo cardiomyopathy have elevated catecholamine blood concentrations. Takotsubo cardiomyopathy was also strongly associated with mental illnesses (19). Although our patient did not undergo angiography, we find takotsubo cardiomyopathy a more likely diagnosis than ST-elevation myocardial infarction for several reasons. The degree of ECG changes observed in the initial ECG, along with a severely reduced left ventricular ejection fraction, would not likely resolve in 24 hours in a patient with conservatively treated ST elevation myocardial infarction. Furthermore, troponin T peak of <2000 ng/L is rarely observed in cases of anterior myocardial infarction, even when timely reperfusion is performed. The patient could have stable coronary disease due to his significant history of nicotine abuse, which might have temporarily worsened ischemia. However, he never complained of chest pain, either before or after the described event. Moreover, it is unlikely that transient spasm would cause such echocardiographic findings (20). Lastly, deep, symmetric T wave inversion and prolonged QTc interval observed in this case are prominent features of takotsubo cardiomyopathy (17).

Most patients with ECT-induced takotsubo cardiomyopathy were women older than 70 treated for major depression (4-7,10,11,14). Due to hormonal changes, post-menopausal women could be more susceptible to cardiovascular events after a sympathetic response to acute stressors, such as ECT application (18). However, men show a greater autonomic response to stress than pre-menopausal women of the same age (21). Psychosis has been associated with catecholamine dysfunction, and excessive psychological stress with long-term norepinephrine dysfunction (22). Therefore, positive psychotic symptoms in our patient could have led to elevated catecholamine blood concentrations.

Grubisha et al (16) reported on a case of takotsubo cardiomyopathy after concomitant ECT and clozapine use, which points to a synergistic effect of ECT and clozapine. Clozapine is a strong antagonist of different subtypes of adrenergic, cholinergic, and histaminergic receptors and systematically elevates epinephrine and norepinephrine levels. Catecholamine hypothesis of ECT-induced takotsubo cardiomyopathy is supported by the burst activity of norepinephrine neurons not only due to ECT, but also due to clozapine and nicotine use, as was shown in the locus coeruleus in an animal model (23). How these therapies interact to alter levels of catecholamine remains unclear. Reports on ECT-induced takotsubo cardiomyopathy show that antidepressants alter the neuronal reuptake of plasma catecholamine and facilitate myocardial stunning by increasing local catecholamine levels (Table 1).

Adrenergic blockade before, during, and after ECT minimizes the risk of takotsubo cardiomyopathy recurrence (5). There are a few patients who underwent successful retrials of ECT after having developed takotsubo cardiomyopathy (6). Takotsubo cardiomyopathy treatment includes beta blockers during the period of ventricular recovery (18), and beta blockers have been shown in animal models to improve left ventricular ejection fraction (24). There are three reports on the concomitant use of beta blockers in patients who develop takotsubo cardiomyopathy, similar to our case (5,7,12). Patients may be predisposed to develop takotsubo cardiomyopathy by conditions that raise baseline catecholamine level (17,19). Both caffeine and nicotine elevate catecholamine blood levels (25), and our patient’s caffeine and nicotine use could have precipitated a cardiovascular accident. In this case, simultaneous effects of clozapine, caffeine, nicotine, and ECT, along with male sex, seem to have overpowered the protective effect of beta blockers on takotsubo cardiomyopathy development.

In conclusion, our case shows that takotsubo cardiomyopathy can occur as a rare complication of ECT in a male psychotic patient, despite the concomitant beta-blockade treatment. Clinical understanding of rare cardiac ECT complications could improve early recognition of patients at risk for takotsubo cardiomyopathy and ensure safe ECT protocols.
